# Identification and Phylogenetic Characterisation of Novel Adeno‐Associated Virus Capsids in Non‐Human Primate Tissues

**DOI:** 10.1111/cpr.70127

**Published:** 2025-09-16

**Authors:** Liyu Zhu, Kai Xu, Yali Ding, Kailun Liu, Jing Liu, Zongren Hou, Rui Niu, Ning Yang, Hualing Qin, Baoyang Hu, Ying Zhang, Wei Li

**Affiliations:** ^1^ State Key Laboratory of Organ Regeneration and Reconstruction Institute of Zoology, Chinese Academy of Sciences Beijing China; ^2^ University of Chinese Academy of Sciences Beijing China; ^3^ Beijing Institute for Stem Cell and Regenerative Medicine Beijing China; ^4^ Medical School University of Chinese Academy of Sciences Beijing China

**Keywords:** adeno‐associated virus, antibody escape, co‐evolution, natural discovery, protein engineering, transduction

## Abstract

Adeno‐associated virus (AAV) has emerged as the predominant viral vector in clinical gene therapy. However, its widespread application confronts critical challenges, including pre‐existing neutralising antibodies in 40%–80% of the population, species‐dependent therapeutic discrepancies, and suboptimal tropism specificity. While current AAV capsid modification strategies (e.g., directed evolution and rational design) have advanced the field, their implementation has been hampered by incomplete mechanistic understanding and persistent translational roadblocks, necessitating the need for the discovery of novel AAV capsids. In this study, we systematically captured 1925 natural AAV variants from non‐human primate (NHP) tissues by integrating multiple Polymerase Chain Reaction (PCR) primers and deep long‐read sequencing technology, significantly expanding the natural capsid library by more than 20‐fold and identifying 1274 representative AAV11 family variants. Based on the co‐evolution analysis of these natural AAV11 variants, we designed the engineered variant AAV11.P5V6, which showed significantly enhanced transduction efficiency in human and NHP primary hepatocytes in vitro and achieved efficient targeting in a mouse central nervous system model. In addition, AAV11 and its variants maintain a strong antibody escape ability in human serum and immune animal models, exhibiting unique serological characteristics with almost no cross‐neutralisation reaction with AAV8 and AAV9, confirming its low serum prevalence and immune evasion advantages. This study established a systematic framework of ‘natural discovery–evolutionary analysis–functional optimization’, providing a new paradigm for the development of next‐generation AAV vectors with clinical‐grade tissue specificity, low immunogenicity, and cross‐species compatibility.

## Graphical abstract

AAV's widespread application is currently confronting critical challenges. This study established a systematic framework of ‘natural discovery–evolutionary analysis–functional optimization’, providing a new paradigm for the development of next‐generation AAV vectors with clinical‐grade tissue specificity, low immunogenicity, and cross‐species compatibility.

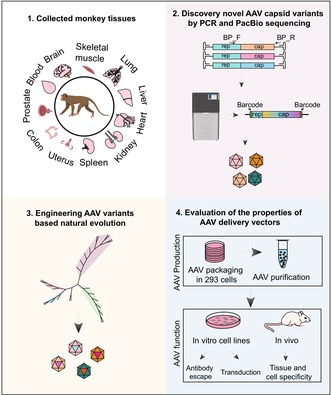

## 1 Introduction

Recent clinical trials have demonstrated the safety and efficacy of adeno‐associated virus (AAV)‐mediated gene therapies owing to its low pathogenicity, minimal integration into the host genome, sustained transgene expression, and broad tissue tropism [1, 2]. However, despite remarkable success in trials for diseases such as spinal muscular atrophy, the clinical translation of AAV therapies faces several significant challenges. The first barrier is the prevalence of pre‐existing neutralising antibodies (NAbs) against wild‐type AAV (wtAAV) in 40%–80% of the population, attributable to prior natural infections [3, 4]. Secondly, high‐dose systemic administration, required to achieve therapeutic protein levels in hepatic disorders, frequently induces dose‐limiting hepatotoxicity [5]. Furthermore, suboptimal targeting efficiency and off‐tissue transduction continue to hinder critical applications, delaying broader clinical adoption in areas such as central nervous system (CNS) disorders and systemic diseases requiring precise organotropism [6]. These challenges underscore the urgent need for the development of novel AAV capsid variants to overcome biological barriers and immune evasion.

Over the past two decades, AAV capsid engineering has emerged as a critical strategy to overcome the limitations of wild‐type vectors and develop capsids with enhanced tissue tropism and functionality [1, 2]. While current AAV capsid engineering approaches such as directed evolution [7, 8, 9, 10, 11, 12] and rational design [13, 14, 15, 16, 17] have significantly advanced viral vector development, their widespread implementation remains constrained by the incomplete understanding of capsid biology and persistent challenges in clinical translation, necessitating the need for discovery of novel AAV capsids with enhanced therapeutic capabilities. Natural discovery represents a complementary approach in the development of AAV capsids, having yielded multiple clinically relevant serotypes including AAV2, AAV5, AAV8, and AAV9 [18, 19, 20, 21] through systematic characterisation of naturally occurring viral isolates. Previous work has shown that only very few natural AAV variants through natural discovery were obtained from non‐human primates (NHPs) and human tissues because of only a pair of primers targeting the AAV2 genome for Polymerase Chain Reaction (PCR) amplification with Sanger sequencing, fragmented next‐generation sequencing (NGS) or low‐depth long‐read sequencing [19, 22, 23]. Therefore, novel approaches are urgently needed to develop more AAV variants as gene therapy delivery vectors.

Here, we used multiple pairs of PCR primers and long‐range third‐generation deep sequencing technology to amplify 1925 new AAV variant sequences from NHP monkey tissues, greatly expanding the library of wtAAV variants isolated in primate by more than 20‐fold. Especially, we identified 1274 phylogenetically distinct AAV11 variants with tropism to the brain and liver of monkeys. Leveraging the co‐evolution information of AAV11 variants, we designed and engineered novel AAV variants that significantly enhanced in vitro transduction efficiency in human and monkey primary liver cells, as well as improved CNS transduction in mice. In addition, the antibody escape ability of AAV11 and its variants was significantly enhanced, with almost no cross‐reaction with AAV8 and AAV9. Through our systematic framework of ‘natural discovery–evolutionary analysis–functional optimization’, we developed next‐generation AAV vectors with clinically relevant tissue specificity, minimised immunogenicity, and enhanced cross‐species compatibility, overcoming current translational bottlenecks in viral vector development.

## 2 Materials and Methods

### 2.1 Animals and Tissues

The tissue samples of *Macaca fascicularis* were procured from the Hainan Primate Laboratory Animal Developing Co. Ltd. and Hainan New Source Biotech Co. Ltd. All animal experiments were performed according to the guidelines for the care and use of laboratory animals established by the Beijing Association for Laboratory Animal Science and approved under the Animal Ethics Committee of the Institute of Zoology, Chinese Academy of Sciences (Approval number: IOZ‐IACUC‐2022‐167). The 8‐week‐old C57BL/6J male mice used for evaluation of the in vivo experiments were purchased from Beijing Vital River Laboratory Animal Technology Co. Ltd.

### 2.2 Extracted DNA and RNA From Tissues

We harvested 322 tissue samples from 31 *M. fascicularis* , including brain, heart, liver, spleen, lungs, kidneys, skeletal muscle, small intestine, colon, and blood of each macaque, plus the prostate of 10 and the cervix of 2 of them. *M. fascicularis* tissues were harvested following standard dissection protocols, with subsequent storage at −80°C until processing. Before nucleic acid isolation, frozen tissues were thawed under controlled conditions on ice. Each tissue specimen was aliquoted in duplicate (approximately 30 mg per aliquot), with one aliquot allocated for DNA extraction (Omega, D3096‐02), and the parallel aliquot subjected to RNA purification (Invitrogen, 12183018A), followed by reverse transcription into cDNA (Promega, A3500).

### 2.3 AAV Genomic DNA Characterisation and Phylogenetic Reconstruction

Nested PCR amplification was performed on cDNA derived from *M. fascicularis* using the Q5 High‐Fidelity 2X Master Mix (New England Biolabs, M0494S). The first round of amplification was conducted under the following thermocycling parameters: 98°C, 1 min; 35 cycles of 98°C, 10 s, 68°C, 20 s, 72°C, 2.5 min; and 72°C, 10 min. A secondary 25‐cycle PCR was implemented by amplicon barcoding. Primer sequences for both amplification rounds are detailed in the Supporting Information. Post‐amplification, products were screened for AAV‐specific sequences, targeting a ~3.1 kb fragment spanning partial rep and full‐length capsid sequences open reading frames. Size‐validated amplicons were gel‐purified (Zymo Research, D4008), pooled in equimolar ratios, and subjected to single molecule real‐time (SMRT) sequencing on the PacBio Revio platform (Annaroad Gene Technology Co. Ltd., Beijing, China). Bioinformatic processing of sequencing reads and maximum‐likelihood phylogenetic tree construction are described in the Supporting Information.

### 2.4 Stereotaxic AAV Injection

Mice were deeply anaesthetised using 1.25% tribromoethanol (i.p., 0.2 mL/10 g body weight). The stereotactic injection coordinates were selected according to Paxinos and Franklin's The Mouse Brain in Stereotaxic Coordinates, 4th edition. Animals were placed on a stereotactic frame (RWD, Shenzhen, Guangdong, China, 68030). A small volume of virus was injected into the hippocampus (relative to bregma: AP –2.30 mm, ML± 1.80 mm, and DV –2.00 mm), at a rate of 0.05 μL/min using a stereotaxic injector equipped with a pulled glass capillary (Stoelting, Wood Dale, IL, USA, 53311). After the injection was complete, the micropipette was held for an additional 10 min before being withdrawn. Animals were allowed to recover from anaesthesia on a heating pad. Three weeks after injection, animals were anaesthetised with 1% sodium pentobarbital, administered intraperitoneally. Secured on a foam board, they then underwent cardiac perfusion. Upon completion, brain tissue was collected for analysis.

### 2.5 Tissue Processing and Cryosectioning

Mouse brain tissues were harvested through standardised transcardial perfusion, post‐fixed in 4% (w/v) paraformaldehyde (PFA; ‌Affymetrix‌) at 4°C overnight. All samples were then dehydrated in 30% (w/v) sucrose solution (‌Sigma‐Aldrich) at 4°C until sedimentation (‌3–6 days‌), embedded in optimal cutting temperature compound (Sakura Finetek, USA)‌ within pre‐chilled moulds, and flash‐frozen in liquid nitrogen‐cooled isopentane to minimise ice crystal artefacts. Serial coronal sections were obtained using a cryostat (Leica CM3050S)‌ at −20°C chamber temperature, with section thickness optimised for tissue type (‌30 μm for brain), and mounted on adhesion microscope slides (CITOTEST)‌ for storage at −20°C prior to immunofluorescence staining.

### 2.6 Immunofluorescence Staining

Immunofluorescence staining of mouse brain section samples was conducted following established protocols [24], with the following primary antibodies: anti‐GFP (Abcam, ab6673, 1:500), anti‐GFAP (Invitrogen, MA5‐12023, 1:500), and anti‐NeuN (Abcam, ab177487, 1:500). Images were captured using a Carl Zeiss LSM880 or an Andor Dragonfly 200 microscope and then processed with Imaris (v10.2). Fiji/ImageJ (v2.8.0) facilitated cellular quantification, followed by GraphPad Prism (v9.2.0) for statistical analysis.

### 2.7 Plasmid Construction

Capsid sequences of AAV11 (GenBank: AY631966.1), AAV10 (AY631965.1), and AAVrh.48 (AY530561.1) were retrieved from the NCBI GenBank database and synthesised de novo as template DNA. Target capsid fragments were amplified using Q5 High‐Fidelity 2X Master Mix (New England Biolabs, M0494S) under optimised thermocycling conditions, followed by agarose gel purification (Zymo Research, D4008). Purified amplicons were ligated into the *cis*‐plasmid backbone (Addgene plasmid, 112862), which had been linearised by sequential digestion with SwaI (New England Biolabs, R0604S) and EcoRV‐HF (New England Biolabs, R3195V), using the seamless cloning kit (CloneSmarter, C5891‐25). Recombinant constructs were transformed into Stbl3 chemically competent *Escherichia coli* (TransGen Biotech, CD521‐02) via heat‐shock methodology. Positive clones were selected following Sanger sequencing validation (GENEWIZ, Suzhou), and high‐purity plasmid DNA was isolated using alkaline lysis coupled with column‐based purification. Mutagenesis protocols for variant construction were implemented as previously described.

### 2.8 Construction of AAV Capsid Mutant Plasmid

*Cis*‐element plasmid was subjected to double digestion using restriction enzymes EcoRV‐HF (NEB, R3195V) and SwaI (NEB, R0604S). The resulting high‐purity DNA fragment served as the backbone for constructing plasmid mutants. KOD polymerase (TOYOBO, KMM‐201) was used to amplify individual fragments containing mutagenic sites via PCR. The PCR cycling conditions were set as follows: initial denaturation at 98°C for 2 min; followed by 35 cycles of 98°C for 10 s, 60°C for 10 s, and 68°C for 1 min; with a final extension at 68°C for 5 min. Assembly of the mutant plasmids was achieved by ligating the mutagenic fragments with the linearised backbone using a seamless cloning method. The ligation products were transformed into Stbl3 chemically competent cells (TransGen Biotech, CD521‐02) to propagate the recombinant plasmids. Positive clones were verified by Sanger sequencing using the dideoxy chain termination method. Sequence alignment and analysis were performed using SnapGene software (V 5.3). Confirmed positive clones were scaled up using a high‐purity plasmid extraction kit. The purified plasmids were aliquoted and stored at −20°C for subsequent use.

### 2.9 AAV Production

AAV vectors were packaged by co‐transfecting HEK293T cells (Procell, CL‐0005) with the following plasmids at a 1:1:1 M ratio: (i) the transgene‐bearing pX602 plasmid (Addgene, 61593) encoding either a reporter gene cassette (scAAV‐CMV‐luciferase‐T2A‐EGFP‐polyA or ssAAV‐GfaABC1D‐EGFP‐WPRE‐pA), (ii) the helper plasmid pAdΔF6 (Addgene, 112867), and (iii) AAV packaging plasmids. Viral particles were harvested 72 h post‐transfection via cell lysis and nuclease digestion. Two purification strategies were employed: crude viral lysates were prepared without further purification for preliminary titration assays; iodixanol gradient ultracentrifugation was performed to isolate high‐purity AAV particles for in vivo applications, as detailed in the prior method [25]. AAV titre determination was described in Supporting Information Materials and Methods, and the primers used to measure titres are listed in Table S3.

### 2.10 Cell Culture

HEK293T (Procell, CL‐0001), Huh7 (Procell, CL‐0120), and U251 (Procell, CL‐0237) cells were cultured in Dulbecco's Modified Eagle Medium (DMEM) (Gibco, 11965084) with 10% foetal bovine serum (FBS) (Gibco, A5669701) and 1% penicillin–streptomycin (Gibco, 15140‐122). AC16 (BeNa Culture Collection, BNCC337712) cells were cultured in Dulbecco's Modified Eagle Medium/Nutrient Mixture F‐12 (DMEM/F12) (Gibco, 11330‐032) with 10% FBS and 1% penicillin–streptomycin. HMC3 (Procell, CL‐0620) was cultured in minimum essential medium (Gibco, 11140076) with 10% FBS and 1% penicillin–streptomycin. Proliferating human hepatocytes (ProliHH) and culture were provided from Gu Qi's Lab; proliferating monkey hepatocytes (ProliMH) thawed and cultured according to Hui's protocol [26].

### 2.11 In Vitro Cell Lines Transduction Efficiency Assay

HEK293T, Huh7, U251, AC16, HMC3, ProliHH, and ProliMH cells were seeded at a density of 8E3 to 1E4 cells per well in white‐walled clear‐bottom 96‐well plates and incubated overnight under standard culture conditions (37°C, 5% CO_2_). AAV vectors encoding the CMV‐driven luciferase transgene were subsequently transduced at multiplicities of infection (MOI) of 1E4 or 1E5 viral genomes per cell, with triplicate wells per experimental condition. Following a 48‐h incubation, luminescence intensity was quantified using the Firefly Glo Luciferase Reporter Gene Assay Kit (Yeasen Biotechnology, 11404ES80) according to the manufacturer's protocol, with signal acquisition performed on a Synergy HT microplate luminometer (BioTek Instruments).

### 2.12 In Vitro NAb Titration

Twenty‐four hours ago, Huh7 cells were seeded at 1E4 per well in white‐walled, clear‐bottom 96‐well plates and incubated overnight. Dilute the virus with an MOI of 1E4. Intravenous human intravenous immunoglobulin (IVIG) (NanYue, S20013005) was serially diluted with heat‐inactivated FBS for six gradients and incubated with each AAV vehicle for 1 h at 37°C. Transduce Huh7 cells in triplicate with the mixture. After 48 h, luciferase activity is determined using a luciferase detection kit. Human serum (mixed human serum, Lablead, 9193; Sigma, S7023) NAb evasion assay is the same as the IVIGs assay. Mice with different AAVs were injected separately with 5E11 vg by tail vein injection, and blood was collected from the orbital venous plexus after 2 weeks and left to rest for 30 min at room temperature. Mouse serum was obtained after centrifugation at 3000 rpm for 20 min at room temperature.

## 3 Results

### 3.1 Isolation and Discovery of Thousands of Natural AAV Capsid Variants in Monkey Tissues

**FIGURE 1:**
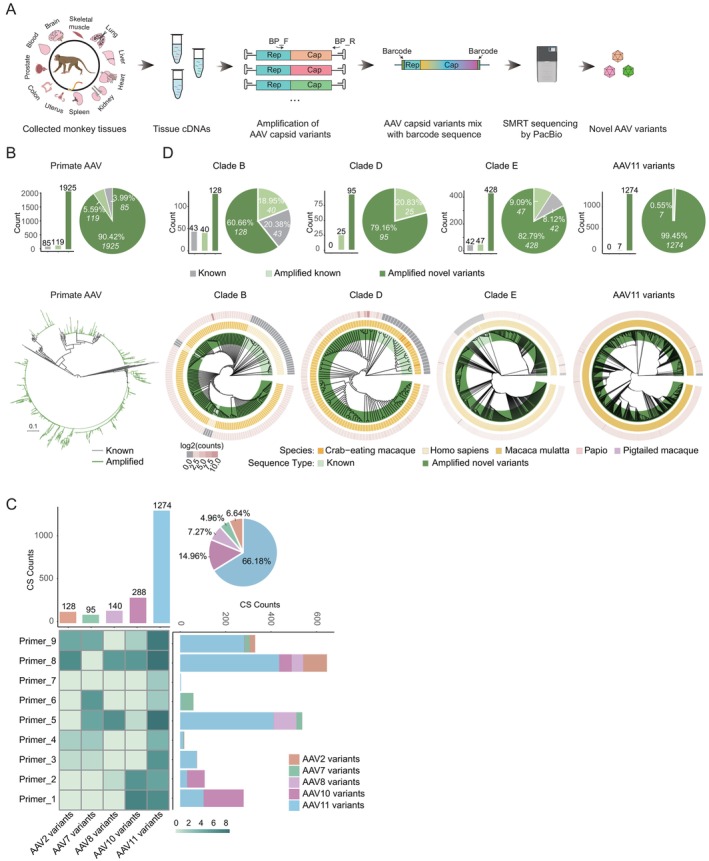
Identification of AAV full‐length capsid sequences from monkey tissues by SMRT sequencing. (A) Workflow for the natural discovery and identification of new AAV variants from monkey tissues. (B) Phylogenetic analysis of all amplified AAV CS DNA sequences compared with known AAV sequences from the database. Sequences identical to known AAVs are shown in light green, known sequences from the database are in grey, and novel amplified sequences are in dark green. The pie chart represents the proportion of these categories. Histograms represent the number of sequences in these categories. (C) Quantification of AAV CS counts obtained through third‐generation sequencing. The upper bar plot shows the total AAV CS count for each variant, while the pie chart represents their relative proportions. The right‐side bar plot illustrates the number of AAV variants amplified by each primer, and the heatmap provides an alternative visualisation of the amplification distribution, where darker colours indicate higher amplification levels. (D) Phylogenetic analysis of clade B, clade D, clade E, and AAV11 variant amino acid sequences compared with known primate AAV sequences. Proportional distribution of clade B, clade D, clade E, and AAV11 variant DNA sequences in comparison to known AAV classifications. The pie chart represents the proportion of these categories. Histograms represent the number of sequences in these categories. The colour scheme follows that of Figure 1B. The outermost ring represents the abundance of each amino acid sequence based on the corresponding DNA sequence count. The middle ring indicates the species origin of each sequence, while the innermost colour‐coded section distinguishes between known sequences from the database (light green) and novel amplified sequences (dark green).

Building upon prior evidence of co‐circulating AAV variants in mammalian tissues [27], we established a systematic workflow to capture naturally evolved AAV diversity through multiplexed primer pairs (Tables S1 and S2) strategically targeting conserved regions across AAV1–13 genomes. Using high‐fidelity Q5 polymerase, we amplified barcoded full‐length capsid sequences from cDNA libraries derived from a comprehensive multi‐organ collection comprising 322 tissue samples from 31 macaques (Figure 1A). High‐throughput long‐read deep sequencing on the PacBio SMRT platform yielded 12,009 distinct AAV VP1 capsid DNA sequences (Figure 1A). To distinguish functional variants from sequencing artefacts, we systematically compared non‐synonymous (dN) and synonymous (dS) mutation rates across sequence identity thresholds (Figure S1). This stringent filtering yielded 1925 full‐length capsid sequences representing bona fide natural mutants, significantly expanding the natural capsid library by over 20‐fold (Figure 1B). Barcode‐enabled primer performance analysis revealed distinct amplification biases across primer sets (Figure 1C), indicating multiple pairs of primers for PCR amplification to discover more novel AAV variants. Maximum likelihood‐based phylogenetic reconstruction revealed no emergence of novel serotypes beyond established evolutionary clades, with all variants clustered exclusively within recognised AAV lineages (Figure 1B). Remarkably, 1274 AAV capsid sequences (66.18% of filtered variants) were classified as AAV11 derivatives—a diversity magnitude vastly exceeding prior reports of AAV11 diversity [28, 29] and representing the largest natural capsid dataset for any single AAV serotype to date (Figure 1D). Notably, a large fraction of these were previously uncharacterized AAV11 variants, thereby substantially diversifying the repository of natural AAV capsids available for therapeutic engineering while preserving native sequence heterogeneity.

### 3.2 Engineering AAV Capsid Variants Based on Co‐Evolution‐Driven Approaches

**FIGURE 2:**
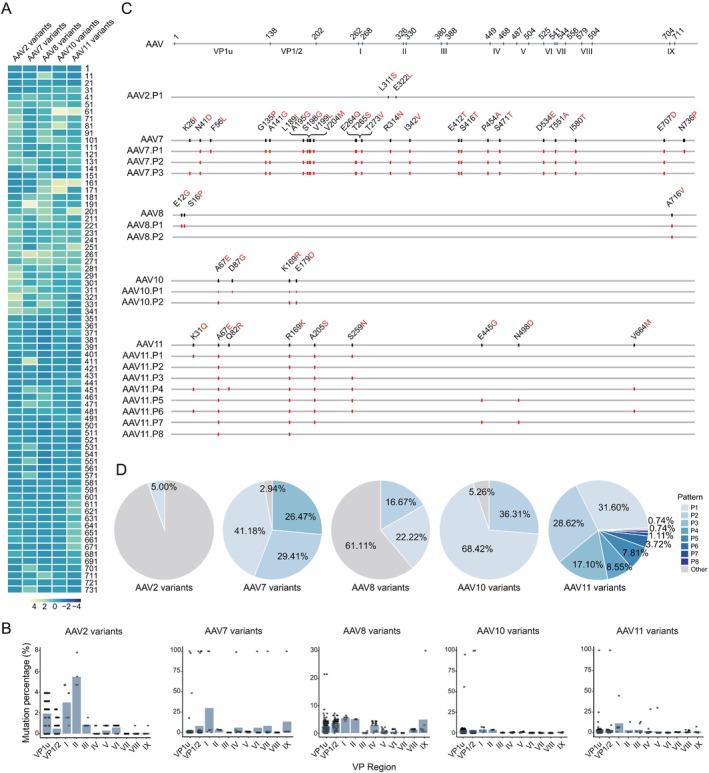
Characterising the evolution of AAV capsid protein and high‐frequency mutation combinations. (A) Mutation frequency distribution of AAV2, AAV7, AAV8, AAV10, and AAV11 variants compared to their respective wild‐type sequences. Mutations were analysed in bins of 10 amino acid positions. The heatmap represents the frequency of mutations at each position, with the colour scale indicating the degree of variation. (B) Mutation percentage distribution across VP regions in AAV variants. Bars indicate the average mutation percentage, while dots represent individual mutation occurrences. (C) Schematic representation of high‐frequency amino acid mutation patterns in AAV variants. Black letters indicate wild‐type amino acids, while red letters denote mutated residues. Mutations are mapped across the VP protein regions for each AAV variant. AAV.Pattern referred to AAV.P, e.g., AAV2 Pattern 1 referred to AAV2.P1. (D) Proportion of high‐frequency mutation patterns in AAV variants. Each pie chart represents the distribution of different mutation patterns within AAV2, AAV7, AAV8, AAV10, and AAV11 variants. Different shades indicate distinct mutation patterns, while the grey section represents other variations. For AAV2, no mutations exceeded the 10% threshold; lowering the cutoff to 5% identified a mutant (L311S, E322L).

Previous studies implicate the nine variable regions in AAV capsids as critical determinants of tropism and immunogenicity [30, 31, 32], with conserved PLA2‐like motifs and nuclear localisation signals identified in VP1/VP2 N‐termini [33]. To investigate whether natural AAV evolution in NHPs has accumulated mutations enhancing infectivity, we analysed features of newly identified capsid sequences. Protein sequence alignment of the capsids of AAV variants within their respective clades revealed non‐uniform mutation frequencies across capsid positions, with elevated mutation rates in specific regions (Figure 2A). This suggested convergent evolution at key sites, prompting us to define high‐frequency mutations as amino acid substitutions occurring in > 10% of variants within a capsid (Figure 2B). Combination analysis of these mutations identified different patterns for various AAV variants (Figure 2C,D, Methods in Supporting Information), and AAV11 variants have the most eight mutation patterns (Figure 2C,D). Interestingly, AAV7.P1–AAV7.P3 naturally evolved two mutations S304N and K217E, which coincide with mutations in AAVRH48R2 by rational design, known to restore the productivity of AAVrh48 (Figure S2) [34]. Subsequently, we constructed an evolutionary tree of these high‐frequency combinatorial variants, illustrating the direction of evolution of each variant (Figure S3).

### 3.3 AAV11 Variants Enhance Transduction in Multiple Hepatocyte and CNS Cell Lines

Given the distinct tissue tropism of AAV variants, we evaluated their enrichment across NHP tissues to guide therapeutic targeting. In addition to highly enriched tissues like the liver and brain, the heart represents another key target for gene therapy. From the perspectives of tissue origin and physiological relevance, the AC16 cell line (immortalised human cardiomyocytes) exhibits characteristic cardiomyocyte properties [35]. This allows AC16 cells to effectively simulate the cardiac physiological environment and cellular characteristics, providing direct physiological relevance for evaluating AAV infection efficiency in myocardial tissue [36]. The Huh7 cell line (derived from human hepatocellular carcinoma) was established from a well‐differentiated tumour in a 57‐year‐old Japanese male [37]. Despite its tumour origin, it retains hepatocyte‐like functions and is widely used in virus infection studies [38], including AAV‐liver infection research [39]. These human‐derived cell lines provide effective in vitro models for assessing AAV tissue tropism and verifying infection efficiency.

**FIGURE 3:**
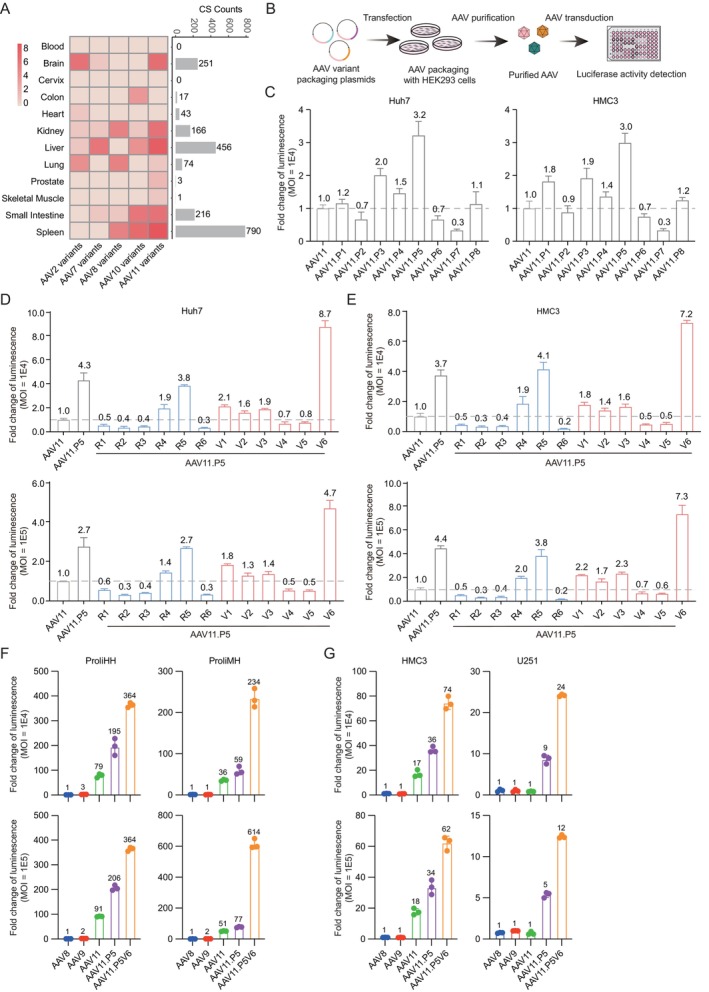
Validation of AAV11 variants in multiple cell lines. (A) The heatmap provides an alternative visualisation of the amplification distribution in each type of monkey tissue, where darker colours indicate higher enrichment for each kind of variant. The right‐side bar plot illustrates the number of AAV variants amplified in every organ tissue. (B) Schematic of AAV in vitro transduction efficiency test. (C) Transduction evaluation of AAV11 high‐frequency mutants in Huh7 and HMC3 at MOI of 1E4. (D, E) Transduction evaluation of AAV11.P5 R series variants (AAV11.P5V1–AAV11P5V6) and V series variants (AAV11.P5V1–AAV11P5V6) in Huh7 and HMC3 at MOI of 1E4 and 1E5. (C–E) Fold difference in luminescence activity from different variants normalised to AAV11. (F) Compared the transduction efficiency of AAV11.P5 and AAV11.P5V6 with AAV8, AAV9, and AAV11 in proliferating human hepatocytes (ProliHH) and proliferating macaque hepatocytes (ProliMH) at MOI of 1E4 and 1E5. Fold difference in luminescence activity from different variants normalised to AAV8. (G) Compared the transduction efficiency of AAV11.P5 and AAV11.P5V6 with AAV8, AAV9, and AAV11 in two nervous system‐associated cell lines (HMC3 and U251) at MOI of 1E4 and 1E5. Fold difference in luminescence activity from different variants normalised to AAV9. (C–G) Data are presented as the mean ± SD (each point represents the mean of three replicates, error bars represent SD), the grey dotted line represents a fold change is one.

To quantify transduction efficiency, we packaged all AAV pattern (AAV.P) variants with a CMV‐luciferase‐GFP cassette and measured luciferase expression in human cell lines. In AC16 and Huh7 cell lines, the high‐frequency pattern variants of AAV7 and AAV10 did not exceed the parents' transduction efficiency by 2‐fold (Figure S4). We noted that AAV11 and its variants exhibited broad tissue distribution, with notable enrichment in brain and liver (Figure 3A). In Huh7 cell lines and HMC3 microglioma cell lines, AAV11.P5 demonstrated ~3‐fold higher transduction than parental AAV11 (Figure 3B,C).

To dissect the functional contributions of individual mutations in AAV11.P5, we employed two engineering strategies: (1) an R‐series of variants in which each AAV11.P5‐defining mutation was introduced into wtAAV11, AAV.P5R1–AAV.P5R6 and (2) a V‐series in which each AAV11.P5 mutation was reverted to the wild‐type residue, AAV.P5V1–AAV.P5V6. Transduction assays of AAV11 engineered variants in Huh7 and HMC3 cell lines revealed that AAV11.P5V6—carrying an asparagine‐to‐aspartate reversion at position 498 (N498D)‐achieved ~2‐fold higher transduction efficiency than AAV11.P5 and a 7‐ to 8‐fold increase over wtAAV11 (Figure 3D,E). At the higher MOI, the transduction efficiency of AAV11.P5V6 remained ~5–7‐fold superior to AAV11 (Figure 3D,E).

Given the limited prior characterisation of AAV11, we compared the transduction efficiency of AAV11.P5 and AAV11.P5V6 with AAV8 for hepatocytes and AAV9 for CNS cells in vitro. Interestingly, in primary human and macaque hepatocytes, AAV11.P5V6 exhibited over 200‐fold higher transduction efficiency than AAV8 and surpassed AAV11 (Figure 3F). Furthermore, in CNS cell lines, AAV11.P5V6 outperformed AAV9 by over 60‐fold in HMC3 and by over 10‐fold in U251 (Figure 3G).

Overall, the engineered AAV11.P5V6 variant demonstrates superior transduction efficiency in primary human hepatocytes and CNS cell types, outperforming benchmark vectors AAV8 for liver and AAV9 for CNS.

### 3.4 AAV11.P5V6 Enhances In Vivo Transduction of the CNS

**FIGURE 4:**
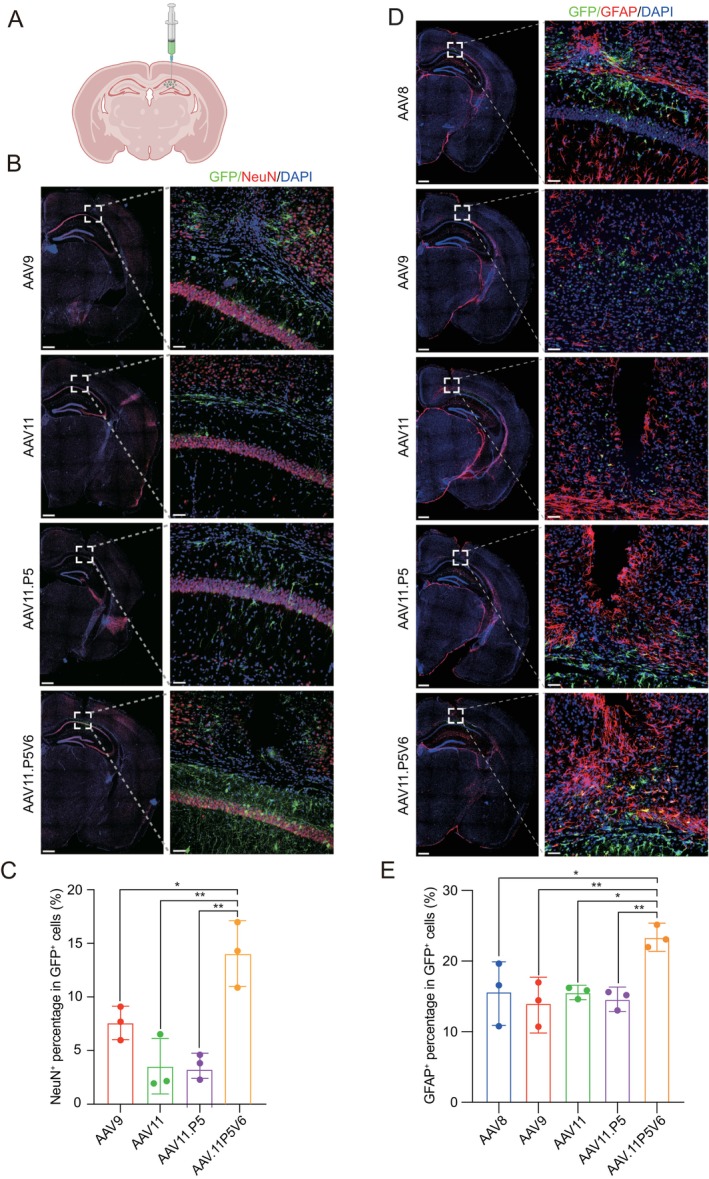
AAV11.P5V6 improves the transduction of the central nervous system in vivo. (A) Schematic illustration of viral injection. Each kind of AAV (3 × 10^9^ vg) was injected into the hippocampal region of C57BL/6 mice (*n* = 3). Schematic created using BioRender.com. (B) Immunofluorescence staining of the hippocampal injection site showing EGFP expression (green) and the neuronal marker NeuN (red). AAV packaged with a CMV‐luciferase‐EGFP cassette. Scale bar = 500 μm (hemisphere section); 50 μm (ROI). (C) Quantification of neuron‐specific transduction in the hippocampal injection site. *p < 0.05; **p < 0.01. One‐way ANOVA. Data are presented as mean ± SD (*n* = 3). (D) Immunofluorescence staining of the hippocampal injection site showing EGFP expression (green) and the astrocyte marker GFAP (red). AAV packaged with a GfaABC1D‐EGFP cassette. Scale bar = 500 μm (hemisphere section); 50 μm (ROI). (E) Quantification of astrocyte‐specific transduction in the hippocampal injection site. *p < 0.05; **p < 0.01. One‐way ANOVA. Data are presented as mean ± SD (*n* = 3).

Previous studies have demonstrated AAV11's ability to transduce neurons and astrocytes in vivo [40]. To evaluate the neuronal transduction efficiency of AAV11 and its variants, we delivered AAVs packaged with a CMV‐luciferase‐EGFP cassette into the hippocampal region of adult mice (Figure 4A). Twenty‐one days after injection, neuronal transduction efficiency was quantified by calculating the percentage of EGFP+ cells co‐localised with the neuronal marker NeuN at the injection site (Figure 4B). Remarkably, AAV11.P5V6 achieved approximately 15% neuronal transduction, nearly double the transduction efficiency of AAV9, while AAV11 and AAV11.P5 reached only ~50% of AAV9's efficiency (Figure 4C). To assess astrocyte‐specific transduction, we utilised a truncated GfaABC1D promoter to drive EGFP expression. Co‐localisation of EGFP+ cells with the astrocytic marker GFAP in the hippocampus revealed that AAV11.P5V6 achieved more than 20% astrocyte transduction, significantly outperformed AAV8, AAV9, and parental AAV11 (Figure 4D,E). Collectively, these findings highlight the superior CNS tropism of AAV11.P5V6 and demonstrate its significant potential for clinical gene therapy applications requiring precise neuronal and astrocytic delivery.

### 3.5 AAV11 Variants Exhibit Significantly Reduced Cross‐Reactivity and Enhanced NAb Evasion

**FIGURE 5:**
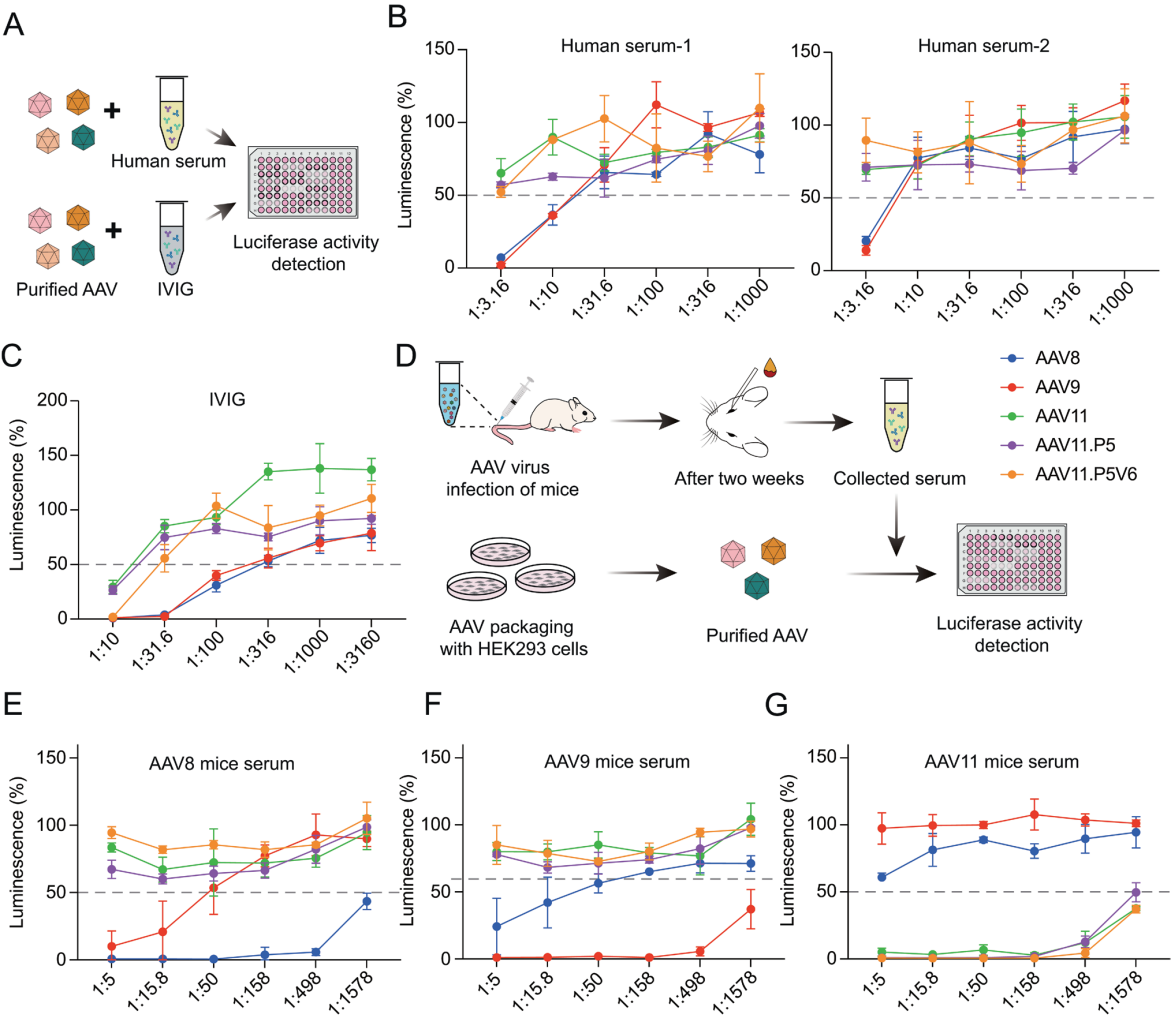
Antibody neutralising activity of AAV11 variants. (A) Schematic of in vitro neutralisation of AAV. (B, C) Neutralised AAV11 variants, AAV11, AAV8, and AAV9 by using human intravenous immunoglobulin (IVIG) and two human serum pools. (D) Workflow for different AAV neutralisation analysis of AAV‐treated mice serum. (E–G) Detected neutralising activity of AAV8, AAV9, AAV11, AAV11P5, AAV11P5V6 with AAV8/AAV9/AAV11‐treated mouse serum. The grey dotted line represents gene‐delivery efficiency to 50% of that in the absence of neutralising antibody. All data points are presented as the mean ± SD (each point represents the mean of three replicates, error bars represent SD).

Pre‐existing NAbs against naturally circulating AAVs remain a major barrier to clinical gene therapy, underscoring the importance of serotypes with low seroprevalence in human populations. We performed in vitro neutralisation assays using pooled human IVIG and two commercial human serum pools to evaluate AAV8, AAV9, AAV11, AAV11.P5, and AAV11.P5V6 (Figure 5A–C). AAV11.P5 and AAV11.P5V6 exhibited sensitivity profiles similar to parental AAV11. High‐dose IVIG (> 40 μg) neutralised 50% of AAV11, AAV11.P5, and AAV11.P5V6 transduction, whereas low‐dose IVIG (~4 μg) reached the neutralisation endpoint for AAV8 and AAV9. Neither serum pool neutralised AAV11 variants, even at the lowest dilution. In contrast, AAV8 and AAV9 were neutralised at dilutions of 1/10 to 1/31.6 (serum pool 1) and 1/3.16 to 1/10 (serum pool 2). These results demonstrated that AAV11 and the engineered variants possess advanced antibody escape capabilities.

To assess in vivo antibody cross‐reactivity, mice were intravenously injected with AAV8, AAV9, or AAV11 (Figure 5D). Serum collected two weeks post‐injection revealed that anti‐AAV8 and anti‐AAV9 sera failed to neutralise AAV11, AAV11.P5, or AAV11.P5V6, even at the maximum dilution (1:1578), while retaining full self‐neutralisation (Figure 5E,F). Anti‐AAV11 sera showed no cross‐neutralisation against AAV8 or AAV9 but potently neutralised AAV11, AAV11.P5, and AAV11.P5V6 at a 1:1578 dilution (Figure 5G). These findings confirm serological divergence between AAV8/AAV9 and AAV11 variants, with AAV11.P5 and AAV11.P5V6 retaining immunological parity with parental AAV11.

## 4 Discussion

As an important vector in the field of gene therapy, AAV still faces clinical application challenges. NAbs can significantly reduce vector delivery efficiency, potentially causing treatment failure [41]. Moreover, insufficient tropism necessitates high‐dose administration, which may trigger immune responses and exacerbate toxicity [6]. To address these limitations, we established a systematic approach to identify AAV variants from NHPs with reduced sensitivity to NAbs and enhanced infection efficiency.

While current AAV capsid engineering strategies such as directed evolution [7, 8, 9, 10, 11, 12, 39] and rational design [13, 14, 15, 16, 17] have advanced the field, their implementation has been hampered by incomplete mechanistic understanding and persistent translational roadblocks, underscoring the need for strategies leveraging natural variant discovery and human‐relevant functional validation. Compared to laboratory‐driven selective pressures for obtaining AAV capsids, natural evolutionary pressures cause capsid sequence to converge at functionally beneficial sites. This convergence enables rapid acquisition of optimised AAV capsid through a discovery, analysis, and engineering transformation. In this study, leveraging the breadth of our newly isolated natural AAV variants, we engineered novel AAV11 mutants through co‐evolution‐guided engineering. Meanwhile, we also demonstrated that AAV11 and AAV11.P5V6 could successfully transduce multiple human hepatocytes in vitro, neurons and microglia of the CNS of mice with high efficiency, indicating the potential as a gene therapy delivery vehicle.

Pre‐existing NAbs against AAV capsids, prevalent in human populations, pose a major clinical barrier to systemic AAV therapy by impairing transduction efficacy, as evidenced by pre‐clinical and clinical studies [42]. Our findings revealed that AAV11 and its derivatives exhibit ≥ 10‐fold antibody escape ability compared to AAV8 and AAV9 in vitro and almost no cross‐reactivity with AAV8 and AAV9 in vitro. These findings validate AAV11's low seroprevalence and distinct antigenicity, positioning it as a clinically advantageous platform to circumvent pre‐existing immunity in gene therapy applications.

Historically, AAV genome characterisation depended on limited primer pairs for PCR amplification with Sanger sequencing [19, 29, 43], inherently lacking resolution for complex viral populations. Although NGS enhanced mutation detection sensitivity, its short‐read lengths required computational assembly of full‐length capsids, generating reconstruction artefacts and obscuring mutation linkage. Third‐generation sequencing revolutionised this paradigm by enabling single‐molecule resolution of complete capsid sequences without assembly [23], yet remained constrained by PCR amplification and low sequencing depth for comprehensive variant discovery. In our work, with the multiple pairs of PCR primers and high‐depth long‐range third‐generation sequencing technology, we amplified 1925 novel AAV variant sequences from 322 tissues of 31 NHP monkeys, especially numerous previously uncharacterised AAV11 variants, greatly expanding the repertoire of natural AAV variants by more than 20‐fold as potential delivery vectors for gene therapy.

Based on the previous problem encountered in directed evolution, species‐specific variation [44], many laboratories have iterated through multiple animal models to produce effective AAV variants with cross‐species compatibility [45]. Although this approach may screen out some species‐specific variants, it remains the fastest and most effective method to generate functional mutants. Naturally discovered AAVs, such as AAV8 and AAV9, were validated through cell lines and mice—currently the most cost‐effective verification strategy.

In our study, AAV11.P5V6 demonstrated transduction efficiency at least an order of magnitude higher than AAV9 in HMC3 and U251 cell lines. However, its transduction efficiency in mouse GFAP‐positive glial cells was less pronounced, showing only a nearly 2‐fold increase. We consider three potential explanations for this disparity. Firstly, when assessed in cell lines in vitro, the MOI can reach 1E4 or 1E5, significantly exceeding typical in vivo MOI levels. Secondly, the cellular environment in vitro is simplified, while the in vivo environment is far more complex. Finally, species differences may contribute to reduced infection efficiency in vivo. As it advances to clinical applications, subsequent studies in NHPs may provide additional insights.

While this study establishes a systematic platform for deep mining and evolution‐guided engineering of natural AAV variants, several areas warrant further refinement to advance translational potential, including comprehensive multiorgan biodistribution profiling (e.g., heart, muscle) with promoter optimisation and in vivo validation of immunoevasion in NHP under physiological stress, alongside transgenic NHP models to resolve species‐specific hepatocyte discrepancies. Integrating artificial intelligence‐driven capsid prediction from natural variants could advance clinical‐grade vectors with precise tropism, minimised immunogenicity, and enhanced translational fidelity.

## 5 Conclusion

In summary, by integrating an enhanced natural capsid discovery pipeline with a high‐depth SMRT sequencing strategy, we pioneered an evolution‐driven AAV discovery platform, uncovering thousands of novel primate‐derived variants and engineering AAV11 mutants with highly effective targeting of human hepatocytes and the CNS of mice, while exhibiting distinct antigenicity (≥ 10‐fold NAb evasion vs. AAV8 and AAV9). This evolutionary decoding strategy redefines natural capsid resource utilisation, bridging biodiversity with clinical vector design to overcome immunity barriers and advance precision gene therapy.

## Author Contributions

Wei Li conceived the idea. Liyu Zhu and Kai Xu designed the experiments. Liyu Zhu performed the experiments. Yali Ding performed bioinformatics analysis. Kailun Liu, Jing Liu, and Zongren Hou helped with the nervous system validation experiment of mice. Rui Niu helped with NAb titration. Wei Li, Ying Zhang, Baoyang Hu, Liyu Zhu, and Kai Xu performed manuscript writing, review, and editing. All authors read and approved the final manuscript.

## Ethics Statement

All animal experiments were performed according to the guidelines for the care and use of laboratory animals established by the Beijing Association for Laboratory Animal Science and approved under the Animal Ethics Committee of the Institute of Zoology, Chinese Academy of Sciences.

## Conflicts of Interest

The authors declare no conflicts of interest.

## Supporting information


**Figure S1:** Distribution of dN/dS values for original and conserved sequences under different similarity thresholds. Histograms show the distribution of nonsynonymous to synonymous substitution ratios (dN/dS) for the original AAV protein‐coding sequence (top left) and conserved sequence (CS) sets defined under varying sequence similarity thresholds (99.8%–97%). CS sequences were compared to the reference AAV sequence to compute the frequencies of conservative and radical amino acid substitutions. The original sequence exhibits a near‐normal dN/dS distribution, while CS sets with similarity thresholds below 99.5% show progressively increased dN/dS ratios, indicating potential shifts in selective constraints. Based on this trend, 99.5% similarity was selected as the threshold for defining CS sequences.
**Figure S2:** Schematic representation of high‐frequency amino acid mutation patterns in AAV7 variants. Black letters indicate AAVrh48 amino acids, while red letters denote mutated residues.
**Figure S3:** Phylogenetic analysis of high‐frequency mutation pattern‐associated AAV amino acid sequences alongside representative known AAV sequences. Different colours indicate distinct AAV types.
**Figure S4:** Different kinds of AAV high‐frequency mutant variants for transduction in cell lines. (A, C) AAV7 and AAV10 high‐frequency mutant variants were transduced with an MOI of 1E4, and the luciferase activity value readings after 48 h in AC16. (B, D) High‐frequency mutant variants of AAV7 and AAV10 have MOI as 1E4, and the luciferase activity value readings after 48 h in Huh7. RLU is a relative fluorescence unit, and the *Y*‐axis represents the fluorescence intensity. The data represent the mean and standard deviation of the three replicates. AAV8.P1 (E12G, S16P) could not be produced.
**Table S1:** Natural discovery of nested PCR first round primers.
**Table S2:** Natural discovery of nested PCR second round primers.
**Table S3:** AAV titre detection primers.

## Data Availability

The raw sequence data reported in this paper have been deposited in the Genome Sequence Archive [46] in National Genomics Data Center [47], China National Center for Bioinformation/Beijing Institute of Genomics, Chinese Academy of Sciences (PRJCA039476), which are publicly accessible at https://ngdc.cncb.ac.cn/.
