# Supplementary material for: Identification and Phylogenetic Characterisation of Novel Adeno‐Associated Virus Capsids in Non‐Human Primate Tissues
Source: Cell Prolif. 2025 Sep 16;59(5):e70127. doi: 10.1111/cpr.70127 (PMC13114770; doi:10.1111/cpr.70127)
Supplement: Supplementary file 1 — Figure S1: Distribution of dN/dS values for original and conserved sequences under different similarity thresholds. Histograms show the distribution of nonsynonymous to synonymous substitution ratios (dN/dS) for the original AAV protein‐coding sequence (top left) and conserved sequence (CS) sets defined under varying sequence similarity thresholds (99.8%–97%). CS sequences were compared to the reference AAV sequence to compute the frequencies of conservative and radical amino acid substitutions. The original sequence exhibits a near‐normal dN/dS distribution, while CS sets with similarity thresholds below 99.5% show progressively increased dN/dS ratios, indicating potential shifts in selective constraints. Based on this trend, 99.5% similarity was selected as the threshold for defining CS sequences. Figure S2: Schematic representation of high‐frequency amino acid mutation patterns in AAV7 variants. Black letters indicate AAVrh48 amino acids, while red letters denote mutated residues. Figure S3: Phylogenetic analysis of high‐frequency mutation pattern‐associated AAV amino acid sequences alongside representative known AAV sequences. Different colours indicate distinct AAV types. Figure S4: Different kinds of AAV high‐frequency mutant variants for transduction in cell lines. (A, C) AAV7 and AAV10 high‐frequency mutant variants were transduced with an MOI of 1E4, and the luciferase activity value readings after 48 h in AC16. (B, D) High‐frequency mutant variants of AAV7 and AAV10 have MOI as 1E4, and the luciferase activity value readings after 48 h in Huh7. RLU is a relative fluorescence unit, and the Y‐axis represents the fluorescence intensity. The data represent the mean and standard deviation of the three replicates. AAV8.P1 (E12G, S16P) could not be produced. Table S1: Natural discovery of nested PCR first round primers. Table S2: Natural discovery of nested PCR second round primers. Table S3: AAV titre detection primers. [file CPR-59-e70127-s001.docx]

**Supplementary information**

**Materials and Methods**

**Sample sequencing**

Long-read sequencing was performed on DNA fragments generated via a custom barcode amplification strategy, enabling unique sample identification during sequencing. Long-read sequencing was performed using a third-generation sequencing platform (Pacbio Sequel IIe), generating raw FASTQ files.

**Data preprocessing and quality control**

**Demultiplexing and read processing**

Raw sequencing reads were demultiplexed based on predefined barcode sequences using a custom script, resulting in 141 individual FASTQ files. This step ensured that each read was assigned to its corresponding sample according to the barcode used during library preparation. The demultiplexed reads were mapped to the wild-type Adeno-Associated Virus (AAV1-13) reference genome using BWA-MEM (v0.7.17-r1188) with default parameters.

**Sequence filtering and quality control**

To improve data quality and reliability, the following filtering criteria were applied: 1. Duplicate Removal: Identical sequences were removed to eliminate PCR amplification artifacts and prevent overrepresentation of specific reads. 2. Length Filtering: Reads with nucleotide lengths between 1800 nt and 2700 nt were retained to ensure the inclusion of near-full-length AAV genomic fragments. 3. Stop Codon Screening: To exclude truncated or non-functional sequences, only reads where the stop codon appeared within the last 10 amino acids (AA) of the translated open reading frame (ORF) were retained.

**Full-Length Cap sequence selection and completion**

Following sequence filtering, full-length Cap (capsid) protein-encoding sequences were identified. Non-full-length sequences were computationally extended using a reference-guided sequence completion approach to obtain full-length structures. This step ensured that incomplete sequences were corrected for downstream analyses. Ultimately, a total of 17,450 high-quality sequences were retained for further study.

**High-Frequency mutation analysis**

**Clustering of DNA sequences**

To classify DNA sequences into known clades, MMseqs2 easy-cluster (v14.7e284) was used with 90% sequence identity (-min-seq-id 0.9) and 85% coverage (-c 0.85). Clustering was performed with full sequence alignment (--cov-mode 0) and 16 threads (--threads 16) to enhance computational efficiency.

**High-Frequency mutation site identification**

Following clustering, we analyzed high-frequency mutation sites within each clade. For each clade, nucleotide variations were detected and statistically assessed to determine recurrent mutation sites that exceeded a predefined frequency threshold.

**Pattern analysis of high-frequency mutations across Clades**

To compare mutation profiles across clades, co-occurrence patterns of high-frequency mutation sites were computed. This analysis facilitated the identification of shared and unique mutational signatures across different clades.

**Consensus sequence calling and phylogenetic analysis**

To ensure the dataset contained relevant sequences, the raw DNA sequences were screened for the presence of known AAV capsid (Cap) sequences. Sequences that matched previously identified AAV Cap genes were retained for further analysis.

**Refined clustering and consensus sequence generation**

Sequences within each clade, identified in the previous clustering step, were further sub-clustered at 95% sequence identity to improve resolution (determined by the dN/dS value). Within each refined cluster, a consensus sequence (CS) was generated to represent the most frequent nucleotide at each position.

**Phylogenetic analysis of consensus sequences**

The consensus sequences (CS) obtained from the refined clusters were translated into amino acid sequences using SeqKit translate (v 2.5.1) with the standard genetic code. The translated protein sequences were subsequently aligned using MAFFT (v7.453) with default parameters to generate a multiple sequence alignment (MSA). A maximum-likelihood phylogenetic tree was constructed using IQ-TREE2 (v 2.2.2.7) with model selection (MFP) and bootstrap support estimation (ultrafast bootstrap and SH-aLRT, 3000 replicates each).

**AAV titer determination**

Preparation of 5 points for the standard curve: Based on the size of the px602-CMV-luc2-T2A-GFP plasmid, it was calculated that a plasmid count of 1 × 10⁸ requires 0.628 ng of DNA. The px602-CMV-luc2-T2A-GFP plasmid was digested overnight with high-fidelity XhoI (NEB, R0146L) restriction endonuclease. After 1% agarose gel electrophoresis (120V, 30 min), the target band of the digestion product was selected for gel extraction. When its concentration was quantified as 1 × 10¹³ copies/mL, its mass concentration was 62.8 ng/μL. The 62.8 ng/μL solution was then subjected to 6 serial gradient dilutions to serve as the standard points for the standard curve.

Viral sample pretreatment: Take X μL of the sample (usually 20 μL), then add 10% X μL of RQ1 RNase-Free DNase (Promega, M6101) and 10% X μL of 10× Reaction buffer (Promega, M6101), mix well, and incubate at 37°C for 1 hour. To stop the reaction, add 10% X μL of Stop solution (Promega, M6101) and incubate at 65°C for 10 min. Add 1/9 X μL of proteinase K (NEB, P8107S), react at 50°C for 1 hour, and then react at 95°C for 10 min. The treated viral samples were serially diluted 10-fold, 100-fold, and 1000-fold with nuclease-free water.

Probe-based qPCR: A 100-reaction system was prepared by mixing 1000 μL of 2× AceQ U+ Probe Master Mix, 20 μL of Primer-F (10 μM), 20 μL of Primer-R (10 μM), 40 μL of 50× ROX Reference Dye 1, 40 μL of probe, 680 μL of nuclease-free water, and 2 μL of samples for each reaction, as Primer-F and Primer-R in Table S3. The standard curve consisted of 5 points, with 3 technical replicates for each point. Each sample was tested with 3 technical replicates, and 4 wells were used as blank negative controls. The qPCR instrument reaction program was set as follows: 37°C for 2 min; 95°C for 10 min; followed by 40 cycles of 95°C for 10 s and 60°C for 45 s.

Standard curve construction: A straight line was generated based on the CT values of the 5 points of the standard curve. After obtaining the standard curve, the number of viruses per unit volume of each sample was calculated using the linear formula.

**Supplementary Figures**

**
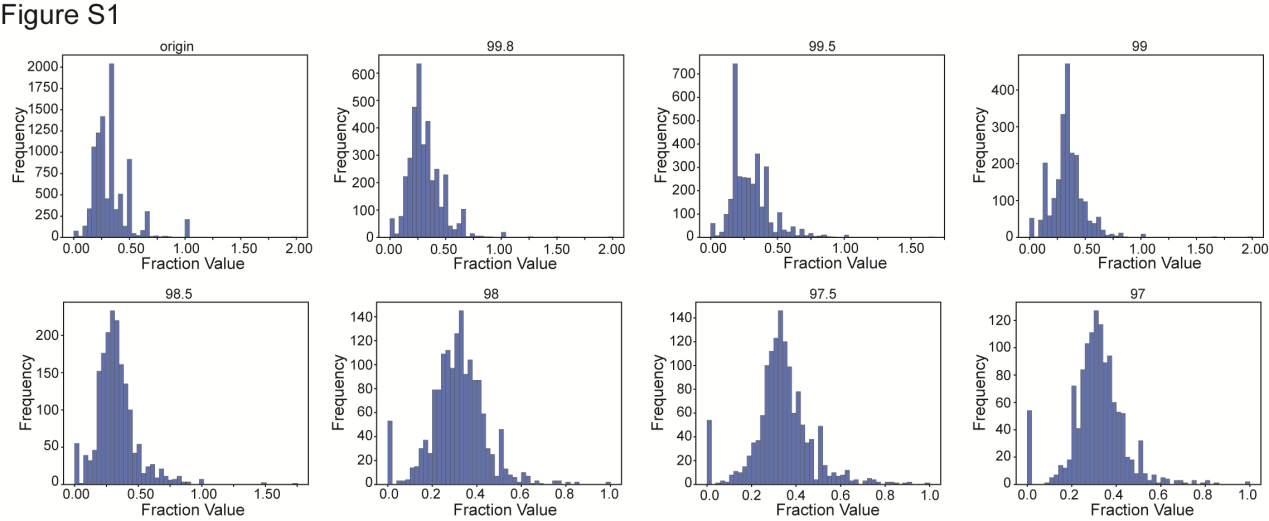
**

**Figure S1.** Distribution of dN/dS values for original and conserved sequences under different similarity thresholds. Histograms show the distribution of nonsynonymous to synonymous substitution ratios (dN/dS) for the original AAV protein-coding sequence (top left) and conserved sequence (CS) sets defined under varying sequence similarity thresholds (99.8% to 97%). CS sequences were compared to the reference AAV sequence to compute the frequencies of conservative and radical amino acid substitutions. The original sequence exhibits a near-normal dN/dS distribution, while CS sets with similarity thresholds below 99.5% show progressively increased dN/dS ratios, indicating potential shifts in selective constraints. Based on this trend, 99.5% similarity was selected as the threshold for defining CS sequences.


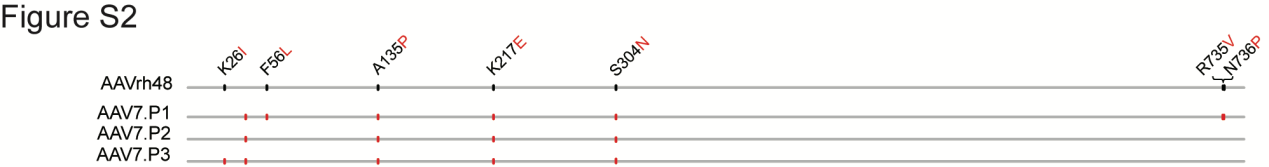


**Figure S****2.** Schematic representation of high-frequency amino acid mutation patterns in AAV7 variants. Black letters indicate AAVrh48 amino acids, while red letters denote mutated residues.

**
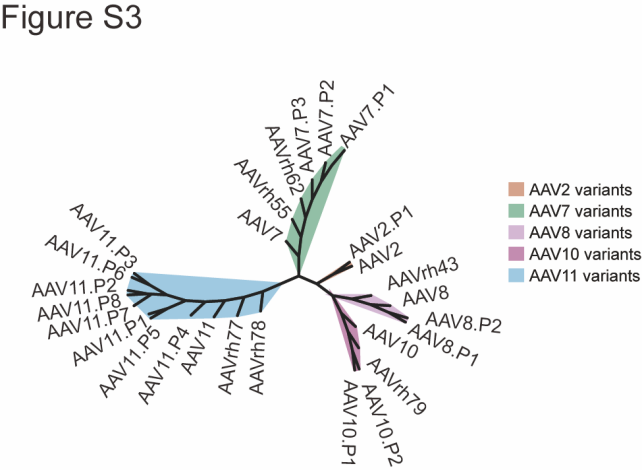
**

**Figure S3.** Phylogenetic analysis of high-frequency mutation pattern-associated AAV amino acid sequences alongside representative known AAV sequences. Different colors indicate distinct AAV types.


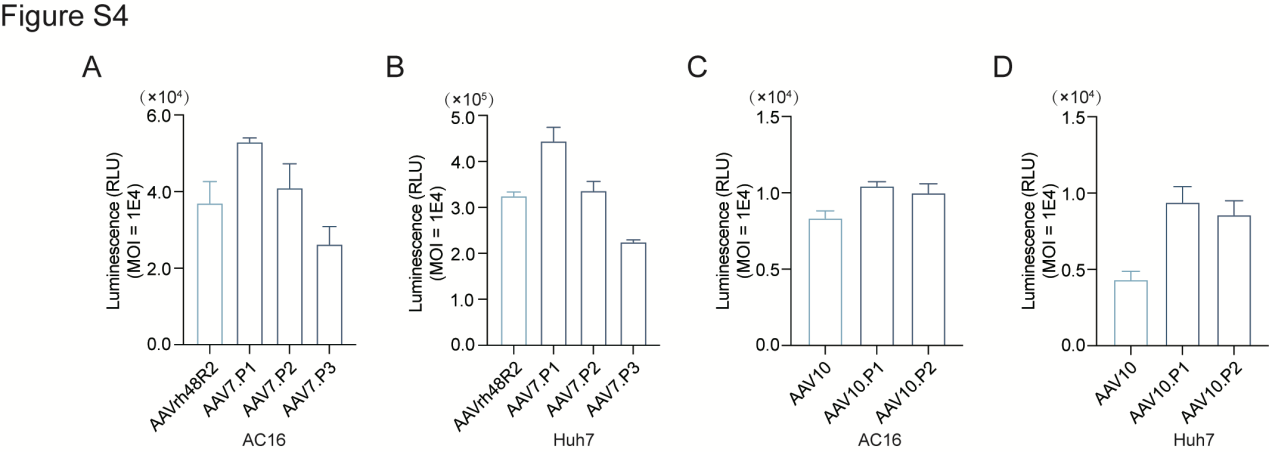


**Figure S****4.** Different kinds of AAV high-frequency mutant variants for transduction in cell lines. (A, C) AAV7 and AAV10 high-frequency mutant variants were transduced with an MOI of 1E4, and the luciferase activity value readings after 48 h in AC16. (B, D) High-frequency mutant variants of AAV7 and AAV10 have MOI as 1E4, and the luciferase activity value readings after 48 h in Huh7. RLU is a relative fluorescence unit, and the Y-axis represents the fluorescence intensity. The data represent the mean and standard deviation of the 3 replicates. AAV8.P1 (E12G, S16P) could not be produced.

**Supplementary Tables**

**Table S1. Natural discovery of nested PCR first round primers**

| **Primer name** | **Primer sequence（5’→3’）** | **Binding AAV DNA sequences** |
| --- | --- | --- |
| Primer_1-F1 | AACTGGACCAATGAGAACTTTCC | AAV1, 2, 7, 8, 9 |
| Primer_1-R1 | AGACCAAAGTTCAACTGAAACGAAT |  |
| Primer_2-F1 | CTGGACCAATGAGAACTTTCCCTTCA | AAV1, 2, 7, 8, 9 |
| Primer_2-R1 | CGCAGAGACCAAAGTTCAACTGAAACGA |  |
| Primer_3-F1 | GTCACCTCCAACACCAACATGTG | AAV11 |
| Primer_3-R1 | TTACAAATGATTAGTCAAATAACGAGAGCC |  |
| Primer_4-F1 | GTCACCTCCAACACCAACATGTG | AAV10 |
| Primer_4-R1 | TTACCTGTCCGGTGCTGTACTG |  |
| Primer_5-F1 | CTGGACCAATGAGAACTTTCCCTTCA | AAV8, 9 |
| Primer_5-R1 | AATCAACCGGTTTATTGATTAACAGGCAATTACA |  |
| Primer_6-F1 | CTGGACCAATGAGAACTTTCCCTTCA | AAV6 |
| Primer_6-R1 | TTACAGGGGACGGGTGAGGTAAC |  |
| Primer_7-F1 | TACGGCTGCGTGAACTGGACCAATGAA | AAV5 |
| Primer_7-R1 | ACGGTTTATTGAGGGTATGCGACATGAA |  |
| Primer_8-F1 | CTGGACCAATGAGAACTTTCCCTTCA | AAV3, 3B |
| Primer_8-R1 | CAACTGAAACGAATTAAACGGTTTATTGATTAAC |  |
| Primer_9-F1 | GTCACCTCCAACACCAACATGTG | AAV1, 7, 8 |
| Primer_9-R1 | GAAACGAATCAACCGGTTTATTGATTAAC |  |
| Primer_10-F1 | CCAATGAGAACTTTCCGTTCAACGATT | AAV4 |
| Primer_10-R1 | ACCGGTTTATTGATTAACAGGTTATTACAG |  |
| Primer_11-F1 | CAAAACAAATGTTCTCGTCACGCG | AAV12 |
| Primer_11-R1 | TTACAAGTGGTGGGTGAGGAAACG |  |
| Primer_12-F1 | CAAAACAAATGTTCTCGTCACGTG | AAV13 |
| Primer_12-R1 | GCAATTACAGATTACGAGTCAGGTATCT |  |

**Table S2. Natural discovery of nested PCR second round primers**

| **Primer name** | **Primer sequence（5’→3’）** | **Binding AAV DNA sequences** |
| --- | --- | --- |
| Primer_1-F2 | GTCACCTCCAACACCAACATGTG | AAV1, 2, 7, 8, 9 |
| Primer_1-R2 | AGACCAAAGTTCAACTGAAACGAAT |  |
| Primer_2-F2 | TTATCTTCCAGATTGGCTCGAGGAC | AAV1, 2, 7, 8, 9 |
| Primer_2-R2 | AGACCAAAGTTCAACTGAAACGAAT |  |
| Primer_3-F2 | CAAAACAAATGTTCTCGTCACGCG | AAV11 |
| Primer_3-R2 | TTACAAATGATTAGTCAAATAACGAGAGCC |  |
| Primer_4-F2 | CAAAACAAATGTTCTCGTCACGCG | AAV10 |
| Primer_5-F2 | GTCACCTCCAACACCAACATGTG | AAV8, 9 |
| Primer_5-R2 | AATCAACCGGTTTATTGATTAACAGGCAATTACA |  |
| Primer_6-F2 | GTCACCTCCAACACCAACATGTG | AAV6 |
| Primer_6-R2 | TTACAGGGGACGGGTGAGGTAAC |  |
| Primer_7-F2 | CTGTCATTGTAACTTCCAATACAAACATGTG | AAV5 |
| Primer_7-R2 | ACGGTTTATTGAGGGTATGCGACATGAA |  |
| Primer_8-F2 | GTCACCTCCAACACCAACATGTG | AAV3, 3B |
| Primer_8-R2 | CAACTGAAACGAATTAAACGGTTTATTGATTAAC |  |
| Primer_9-F2 | CAAAACAAATGTTCTCGTCACGCG | AAV1, 7, 8 |
| Primer_9-R2 | GAAACGAATCAACCGGTTTATTGATTAAC |  |
| Primer_10-F2 | CCAATGAGAACTTTCCGTTCAACGATT | AAV4 |
| Primer_10-R2 | ACCGGTTTATTGATTAACAGGTTATTACAG |  |
| Primer_11-F2 | CAAAACAAATGTTCTCGTCACGCG | AAV12 |
| Primer_11-R2 | TTACAAGTGGTGGGTGAGGAAACG |  |
| Primer_12-F2 | CAAAACAAATGTTCTCGTCACGTG | AAV13 |
| Primer_12-R2 | GCAATTACAGATTACGAGTCAGGTATCT |  |

**Table S3. AAV titer detection primers**

| **Primer name** | **Sequence (5'****→3')** | **Probe modification** |
| --- | --- | --- |
| ITR-titer-F | GGAACCCCTAGTGATGGAGTT | 5' 6-FAM; 3' MGB |
| ITR-titer-R | CGGCCTCAGTGAGCGA |  |
| ITR-titer-P | CACTCCCTCTCTGCGCGCTC |  |
| GFP-titer-F | TCCGCCACAACATCGAGGAC |  |
| GFP-titer-F | GTAGTGGTTGTCGGGCAGCA |  |
| GFP-titer-F | CAGCGTGCAGCTCGCCGACC |  |
